# Towards a 3D-Printed Millifluidic Device for Investigating Cellular Processes

**DOI:** 10.3390/mi15111348

**Published:** 2024-10-31

**Authors:** Jared A. Engelken, Tobias Butelmann, Fabian Tribukait-Riemenschneider, V. Prasad Shastri

**Affiliations:** 1 Institute for Macromolecular Chemistry, University of Freiburg, 79104 Freiburg, Germany; jared.engelken@makro.uni-freiburg.de (J.A.E.); fabian.riemenschneider@makro.uni-freiburg.de (F.T.-R.); 2 BIOSS Centre of Biological Signaling Studies, University of Freiburg, 79104 Freiburg, Germany

**Keywords:** 3D printing, stereolithography, millifluidics, cancer research, metastasis, organ-on-a-chip, AI-enabled design

## Abstract

Microfluidic devices (µFDs) have been explored extensively in drug screening and studying cellular processes such as migration and metastasis. However, the fabrication and implementation of microfluidic devices pose cost and logistical challenges that limit wider-spread adoption. Despite these challenges, light-based 3D printing offers a potential alternative to device fabrication. This study reports on the development of millifluidic devices (MiFDs) for disease modeling and elucidates the methods and implications of the design, production, and testing of 3D-printed MiFDs. It further details how such millifluidic devices can be cost-efficiently and effortlessly produced. The MiFD was developed through an iterative process with analytical tests (flow tests, leak tests, cytotoxicity assays, and microscopic analyses), driving design evolution and determination of the suitability of the devices for disease modeling and cancer research. The design evolution also considered flow within tissues and replicates interstitial flow between the main flow path and the modules designed to house and support organ-mimicking cancer cell spheroids. Although the primary stereolithographic (SLA) resin used in this study showed cytotoxic potential despite its biocompatibility certifications, the MiFDs possessed essential attributes for cell culturing. In summary, SLA 3D printing enables the production of MiFDs as a cost-effective, rapid prototyping alternative to standard µFD fabrication for investigating disease-related processes.

## 1. Introduction

Disease modeling is an essential tool in pathology, oncology, and drug development. Traditionally, experimental approaches have been based on 2D cell cultures *in vitro*, complemented by *in vivo* animal models [1,2,3,4,5,6,7]. However, these methods have various limitations [2,6,7,8,9]. For example, 2D cultures lack accurate *in vivo* features, such as natural barriers, hypoxic gradients, proper tissue stiffness, and cell–cell/matrix interactions, which impairs drug diffusion and reduces their ability to model the human environment [2,7,8,9]. Additionally, animal models cannot accurately predict human drug toxicity and side effects due to species differences [6,9]. It has, therefore, become necessary to develop a reliable, pragmatic, efficient, and cost-effective *in vitro* culture model that closely mimics the *in vivo* microenvironment [5,10,11,12].

To address this need, researchers have developed 3D culture models, such as multicellular spheroids produced with a hanging drop method [13], to mimic tumor-cellular organization and the microenvironment, like the STEMs by Li et al. [14]. The objective of these 3D-cell culture systems is to better emulate the cellular microenvironments of tumors, organs, and the cellular interactions within [5,6,7,15,16,17]. Efforts to replicate the in vivo environment have also led to the establishment of microfluidic device (µFD, <0.5 mm) and millifluidic device (MiFD, >0.5 mm) platforms that combine cell culture with fluidics [2,4,6,11,16,18], which could help create personalized medicines [19]. Such fluidic devices, often termed organ-on-a-chip (OOC), focus on emulating the spatial relationships between various organs and aim to replicate the complex and dynamic interactions between tissues, including physiological flow, shear stress, and nutrient delivery, which are essential for assessing drug metabolism, pharmacokinetics, and cancer metastasis [9]. Both systems require less reagent or medium, generate less waste, are highly portable, and offer easy integration and automation [18,20,21,22], making them superior to traditional, static 2D and 3D platforms [17] and emphasizing physiological readouts using cell metabolic products. A highly simplified system to study cell migration based on such an approach has also been reported as the so-called metastasis-on-a-chip [2,10]. However, such systems often rely on a 2D monolayer cell culture and fail to recognize cellular processes, since metastasis involves the collective migration of cells that require juxtacrine signaling [14]. Furthermore, cells communicate with one another in 3D space through signaling gradients [23], which cannot be replicated without incorporating 3D multicellular constructs with fluidics. This significant aspect is further compounded by the fact that µFDs, due to their dimensional constraints, are not ideally suited to integrate spheroids or tissue-engineered constructs.

Furthermore, from a manufacturing standpoint, the fabrication of µFDs poses some challenges [20,24,25,26]. The standard production methods include micromachining, micro-milling, hot embossing, and injection molding. However, these processes can be imprecise, expensive, less amenable to design changes, take a long time, and often require special processing facilities such as cleanrooms [18,24,25,26,27]. More recent methods include photolithography and soft lithography of polydimethylsiloxane (PDMS) [18,22,26,27,28,29,30], both of which involve a multi-step process of etching and bonding [16,26,28], which is labor-intensive, costly, and does not readily accommodate design changes [9,24,25,26]. PDMS is often preferred for µFDs due to its optical transparency and biocompatibility, but it can swell with nonaqueous solvents and adsorb hydrophobic compounds, leading to experimental errors and limiting its application in solutions composed of biological samples [9,24,27,28,29,31]. These methods are also labor-intensive and limited to 2D geometries, thus making them impractical for large-scale production and high-throughput research [16,24,25,26,28].

To overcome these limitations and expand the utility of fluidics-based in vitro models in the investigation of cellular processes, there is a strong need for a simpler, more efficient, and cost-effective fabrication method for small-scale fluidic devices that supports various materials [24,25], rapid prototyping, and seamless transitions between design, production, and research [9,26]. In response, 3D printing is emerging as a potentially revolutionary solution, especially for small-scale fluidics and on-chip devices [21,24,25,26,32,33,34]. It enables quick modifications, easy reprints, and even automated fabrication, bypassing the drawbacks of micromachining and forming new molds [21]. A further benefit over costly traditional production methods is that 3D printing allows for complex custom designs [21,24,33,35]. Its affordability, portability, and ease of design transfer also promote efficiency, decentralized research, and global collaboration, making advanced analysis platforms accessible even in developing countries [24,26,32,33].

**Figure 1:**
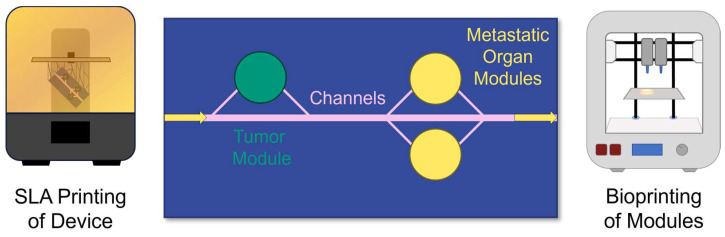
Conceptualization of the MiFD for metastasis research. Top view of channels (pink) connecting a tumor (green) with metastatic organ modules (yellow). Flow direction indicated by arrows. The device is printed with SLA and the modules are bioprinted.

This study demonstrates the feasibility of stereolithographic (SLA) printing to produce a custom device that integrates sub-millimeter-sized conduits for fluid delivery with interchangeable bioprinted modules for defining and accessing tissue and cellular microenvironments, as conceptualized in Figure 1. Furthermore, design variables are elucidated, and the device’s intended function is validated using computational fluid dynamics (CFD) and experimental data to support the device design criteria. Finally, a case is made for developing cytocompatible resins for printing such devices.

## 2. Materials and Methods

### 2.1. Computer-Aided Design (CAD) and CFD

The devices were designed using Inventor Professional 2022–2024 (Autodesk, San Francisco, CA, USA). The finalized designs were readily exported as STL files for 3D printing or kept in their IPT file format for flow simulations with Autodesk CFD v24.1 (Autodesk, San Francisco, CA, USA). CFD analyses were run for 100 iterations with 0.2 mm/min flow at the inlet, 0 Pa at the outlet, and water as the fluid. No specific fluid transport model was applied.

### 2.2. SLA and Post-Processing

A Form 3 stereolithography printer (Formlabs Inc., Somerville, MA, USA) was used to carry out all of the SLA printing. Formlabs BioMed Clear and 3Dresyns OD Clear BIO resins were used in this study. STL files were sliced in PreForm (2024, v3.37.2.361, Formlabs Inc., USA) and printed with the following settings: layer thickness of 0.025 mm, Clear V4 selected resin type, lengthwise rotation of 45°, mini rafts, support with 0.35 mm touch points, and no internal supports. Similar washing, cleaning, and curing procedures were utilized for both resin types, according to the Formlabs printing guidelines [36]. A pressurized air pistol and injected IPA were utilized to flush the channels thoroughly of uncured resin according to the Formlabs white paper on SLA 3D printing for desktop millifluidics [37].

Once the components were fully cured, the remaining support material was removed and discarded. When necessary, the devices were sanded with 100, 240, 500, 1000, and 2000 grit sandpaper and then polished with Burnus acrylic glass polishing paste and a fine cotton cloth to smooth the surface until it appeared transparent and glassy. When top-down microscopy was necessary, the top of the part was painted with uncured resin and then cured again.

### 2.3. Q-Sert Fabrication

The Q-serts were designed with Inventor Professional 2022 software (Autodesk, San Francisco, CA, USA) and were fabricated from Polylactic acid (PLA) filament (2.85 mm; Filamentworld, Neu Ulm, Germany) for 96-well plates using a LulzBot Mini 3D printer (FAME 3D, Fargo, ND, USA) [15]. Before their use in cell culture, the Q-serts underwent sterilization by immersion in 70% ethanol, followed by 30 min of UV irradiation in a laminar flow hood.

### 2.4. Cell Culture

A549-RFP and NIH/3T3 cells were cultured at 37 °C under 5% CO_2_ in Dulbecco’s minimal essential medium (DMEM) with 10% fetal bovine serum (FBS).

#### Spheroid Production

Cell spheroids were produced, as described by Butelmann et al. [15]. Briefly, Q-serts were inserted into the wells of a 96-well plate, and a 35 μL drop of cell suspension (10,000 cells per drop) was pipetted into each Q-sert hanging drop chamber and allowed to aggregate. After 2 days, the medium was exchanged daily by removing 5 μL and adding 6 μL to account for evaporation. To harvest the spheroids, 75 μL of Dulbecco’s Phosphate-Buffered Saline (DPBS) were pipetted through each Q-sert cavity. The Q-serts were then removed, and the spheroids were collected.

### 2.5. MiFD Setup

The fabrication of the MiFD system began with the printing and subsequent post-processing of the device, caps, and module molds. All necessary components, including 3.15 m of tubing, were steam sterilized at 121 °C. The tubing connected the device to the pump with two 1.5 m sections of silicone tubing (2 mm inner-diameter × 6 mm outer-diameter, Schlauch24) and a 15 cm-long section of L/S 14 PharMed PBT—Saint Gobain tubing (1.6 mm inner-diameter × 5 mm outer-diameter, Masterflex, Gelsenkirchen, Germany) connected in the middle with Luer-Lock connectors. The millifluidic system was assembled by connecting the MiFD with the tubing under a biological safety hood, after which the system was flooded with sterile DPBS to remove air and bubbles using a peristaltic pump (2017, iGEM Team RWTH Aachen University) [38]. Meanwhile, the relevant module inserts were prepared, as described in Section 2.5.1, and were gently placed in the device modules. O-rings were positioned on top, and the caps were securely fastened over the modules.

#### 2.5.1. Module Inserts

Two variations of interchangeable inserts were produced: spheroid-laden and fluorescein isothiocyanate (FITC)-dextran-laden. Sterile agarose (10% carboxylated agarose at 60% carboxylation and 90% native agarose) was dissolved at 90 °C and held in a liquid state at 60 °C while the necessary additives were prepared.

Spheroids were individually extracted from their hanging drops and pipetted into the agarose below 40 °C. The spheroid-agarose mixture was then vortexed, and 20 μL were extracted, from which 11 μL were pipetted into the module forms to solidify further.

The fluorescent inserts were prepared by thoroughly mixing 250 μL of sterile agarose with 2.63 mg of FITC-dextran powder (CAS-No. 60842-46-8, Sigma–Aldrich, Co., St. Louis, MO, USA). Once mixed, 11 μL of the fluorescent agarose were pipetted into the module forms to solidify.

### 2.6. Flow Test

The flow behavior within the main channel and modules was visualized using FITC-dextran. Fluorescent inserts, prepared as described in Section 2.5.1, were placed in the module chambers, and the MiFD system was assembled per the procedure in Section 2.5. The MiFD was positioned on a Zeiss Axio Observer Z1 microscope with an AxioCam MRm camera. The flow rate was set to 0.2 mL/min, and images of the channel inlets, outlets, and module regions were captured every 30 min to observe flow dynamics and interactions within the system.

### 2.7. UV/Vis Spectrophotometry

UV/Vis spectrophotometry measurements were carried out to investigate potential cytotoxic leachates. After post-processing and autoclaving, an MiFD was submerged in 30 mL of sterile distilled water for 14 days. The water was subsequently evaporated. The extractant was dissolved in isopropanol and measured with a NanoDrop 2000c spectrophotometer (Thermo Scientific, Waltham, MA, USA) in a quartz crystal cuvette. Uncured resin was also measured in isopropanol. Finally, a Dremel tool with a metal sanding tip was used to grind a fully post-processed print into powder, which was then suspended in isopropanol at 12.5 mg/mL. The supernatant was then measured after letting larger particles settle out.

### 2.8. Perfused-Medium Leaching & MTT Assay

**Figure 2:**
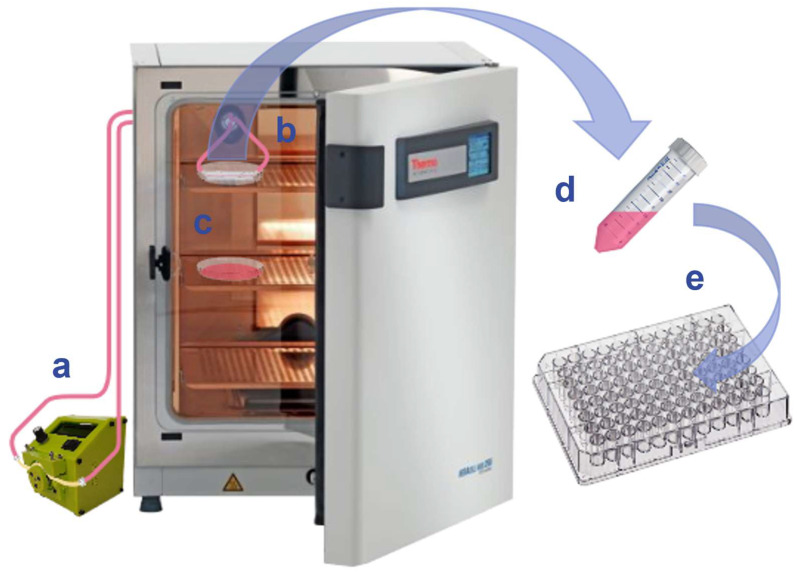
Diagram of the perfused-medium leaching & MTT assay. (**a**) Peristaltic pump. (**b**) MiFD with medium perfusion. (**c**) Control medium. (**d**) Perfused medium extracted. (**e**) 96-well plate for the MTT assay of the perfused medium.

An MiFD was assembled with the necessary tubing, as described in Section 2.5. The peristaltic pump was set to a rate of 0.1 mL/min. A diagram depicting the layout and progression of the experiment is provided in Figure 2. After pumping the medium through the MiFD system for 7 days, the medium was pumped into a sterile, 15 mL centrifuge tube, and a dilution array was prepared. Following the ISO 10993-5 standard protocol for cytotoxicity testing [39], the sample medium was tested against a positive control, a negative control, and their respective medium blanks using an MTT assay with NIH/3T3 fibroblasts. An additional MTT assay was performed after treating a device by sequentially flushing it with isopropanol for 1 h, 0.5% Tween-20 for 12 h, and DMEM (10% FBS) for 24 h, using a peristaltic pump at a flow rate of 0.23 mL/min.

### 2.9. Microscopy

Cells and channels were visualized using a ZEISS Observer A1 (Carl Zeiss, Oberkochen, Germany), a ZEISS Axio Observer Z1 (Carl Zeiss, Oberkochen, Germany), or an Echo Revolve 4K microscope (Echo, San Diego, CA, USA). When applicable, images were analyzed and edited using ImageJ (version 1.53c, NIH, USA).

### 2.10. Atomic Force Microscopy

Scans were performed in tapping mode with a Dimension V atomic force microscope (Bruker Ltd., Billerica, MA, USA) equipped with the Nanoscope software (V.7.3; Bruker Ltd., Billerica, MA, USA). The surface samples were measured with a phosphorus-doped silica cantilever in air (k = 3 N/m, f_0_ = 74–90 kHz) at a scan rate of 0.5 Hz with 256 lines per image. Per condition, five different sample regions with a size of 2 µm² were measured each. NanoScope Analysis (V.1.40; Bruker Ltd.) was used for data analysis and visualization.

### 2.11. Scanning Electron Microscopy (SEM)

Samples were mounted on a conductive carbon tape and sputter coated with gold for 60 s. The coated samples were imaged using an FEI Quanta 250 FEG scanning electron microscope. The images were acquired at an accelerating voltage of 20 kV under soft vacuum (100 Pa) at different magnifications with a large field secondary electron detector.

## 3. Results

### 3.1. CAD & SLA Printing

**Figure 3:**
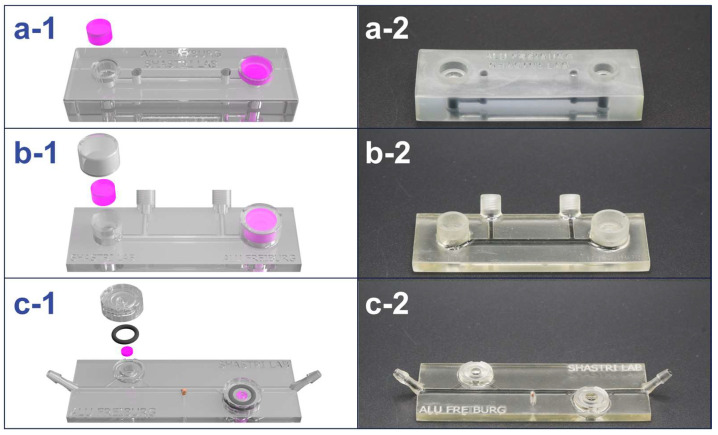
Design evolution of the MiFD with digital renders and photos of selected iterations for better functionality. (**a-1**/**a-2**) First iteration and initial large concept. (**b-1**/**b-2**) Selected model from the intermediate stage of the design process. (**c-1**/**c-2**) Final design, optimized for interstitial flow at the modules with a sample port in the middle. The renders (**a-1**, **b-1**, and **c-1**) depict gel modules (pink), O-rings, and caps, with respect to each design. The channels in the photos (**a-2**, **b-2**, and **c-2**) were filled with an activated charcoal dispersion for better visibility.

The design of the MiFD went through several phases of evolution throughout its development process. An iterative method was used in the MiFD’s development, with feedback from CAD, CFD analysis, flow tests, and leak tests. A selection of the model progression is shown in Figure 3.

#### 3.1.1. MiFD Design

**Figure A1:**
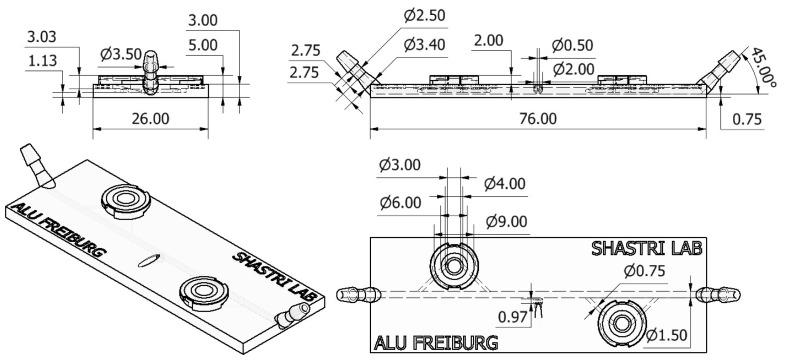
Annotated drawing of the MiFD in its latest iteration. Dimensions presented in mm.

**Figure A2:**
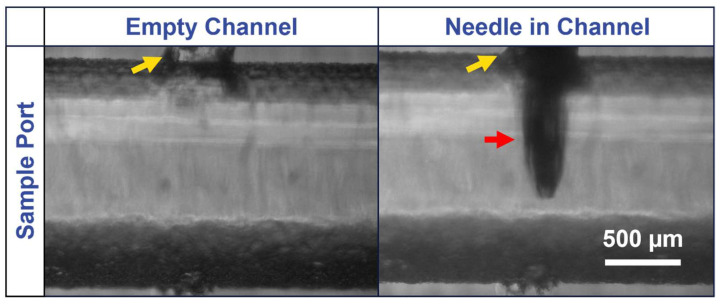
Sample port on the MiFD with a needle sampling. Yellow arrow indicating sample port location, red arrow indicating 30G needle.

The latest iteration of the MiFD development was a culmination of design ideas to optimize efficient production and ease of use, with attention given to flow and functionality. Special consideration was also given to experiment preparation and test applications. These features include a microscope slide design with convenient tubing connections, straightforward twist-on caps, and a sample port. The dimensions of the latest MiFD iteration are provided in Figure A1. It was found that initially flooding the device with the desired fluid was the most effective way to remove air bubbles. After the system was flooded, the gels could be readily slipped into the modules with the caps fitting securely on top. Sterile samples could be extracted through the rubber stopper in the sample port with a 30-gauge needle and syringe, as can be seen in Figure A2. Test devices were designed and fabricated to assess leakage and sampling, leading to the final design (Figure 3c-1,c-2). A series of sterilized devices was evaluated, confirming that the sampling ports and caps remained watertight for at least 7 days, with no detectable signs of contamination.

#### 3.1.2. Printing

A layer height of 0.025 mm was found to be a necessity for achieving the finer details of the caps and modules and in the small channel sizes in the MiFD. At higher layer thicknesses, features below 1 mm in size began to have relatively significant deviations from the CAD model dimensions. For narrow and long cavities, such as the channels of the MiFD, it was found that angling the print 45° lengthwise to the print bed was optimal to minimize clogging and reduce visible layer lines. For an average MiFD, the printing time was around 12 h. The dimensional errors in external features compared to the design models was consistently below 1%, except for the overall device thickness before post-processing, which was up to 12% thicker than the original model, due to its orientation to the print platform. Dimensional errors in the channels did not exceed 5% after post-processing.

#### 3.1.3. Post-Processing

After printing, the components underwent washing and curing to remove excess resin and to further solidify them, as outlined in Section 2.2. For tighter cavities, such as the MiFD channels, a pressurized air pistol and injected IPA were required to flush the channels thoroughly. Once fully cleaned, and when no residual liquid resin could be visually detected, the parts were placed in a Form Cure machine. In addition to further polymerizing the resin and hardening the parts, the curing process decreased the material’s ductility. The applied heat also caused the resin to develop a yellow tint, which intensified after autoclaving. Furthermore, curing occasionally induced slight warping in the MiFD, causing the ends to bend upward. These adverse effects were more pronounced with the 3Dresyns OD Clear BIO resin compared to the Formlabs BioMed Clear resin.

**Figure A3:**
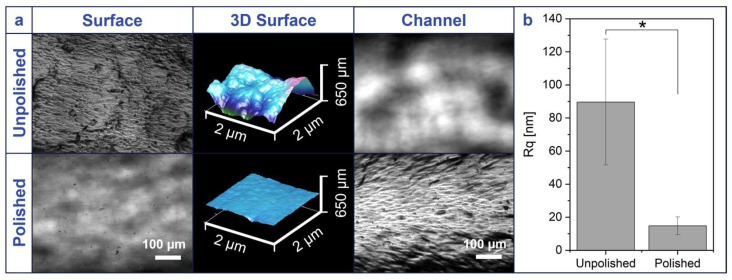
Atomic force microscopy of (un)polished MiFDs. (**a**) Brightfield imaging of the surface and interior of the channel, with 3D surface images by AFM. (**b**) Roughness (Rq) data comparison of unpolished and polished MiFD surfaces. Statistics with n = 5 each, * *p* < 0.05 significant difference between unpolished and polished roughness (Rq) in a Student’s *t*-test.

Despite printing with a layer height of 0.025 mm, all parts retained surface imperfections, usually along layer lines. Without polishing, the flat surface of the MiFD appeared rough and inhibited internal microscopy of the channels, as can be seen in Figure A3. Sanding and polishing the surface, as described in Section 2.2, most efficiently yielded the clearest surface, as depicted in the same figure. Additionally, the difference in roughness was quantified with atomic force microscopy (AFM), as also shown in Figure A3. The AFM data (Figure A3b) confirms that the unpolished surface has a statistically significant variance in topography (Rq = 89.64 ± 38.04 nm) in contrast to the polished surfaces, which have a relatively small variance in elevation (Rq = 14.77 ± 5.38 nm).

### 3.2. Fluid Dynamics

**Figure 4:**
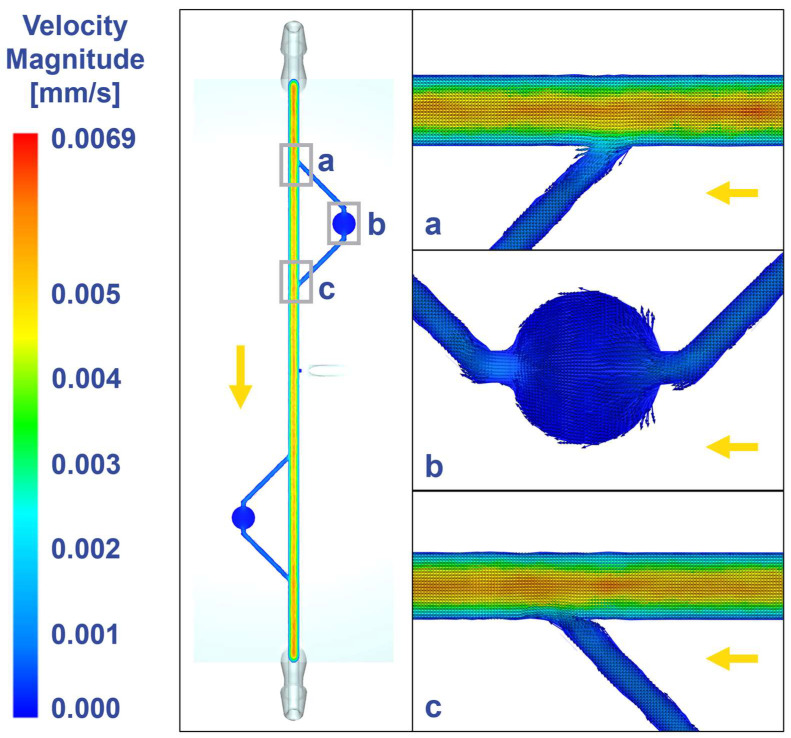
Computational fluid dynamics at different locations of the device revealing interstitial flow at the modules. (**a**) Inlet to the module. (**b**) Module and connecting channels. (**c**) Outlet from the module. Gray rectangles indicate imaging positions, yellow arrow indicates flow direction. Visualization with velocity vectors within the flow plane.

The MiFD design was analyzed with CFD, as shown in Figure 4, allowing for adjustments in flow patterns to achieve interstitial flow velocities [40,41,42] within the modules, which is important for mimicking the physiological flow of tissue microenvironments. The final design maintains steady laminar flow in the main channel (Figure 4a,c) and achieves interstitial flow in the channels leading to, within, and from the modules (Figure 4b).

### 3.3. Flow Tests

**Figure 5:**
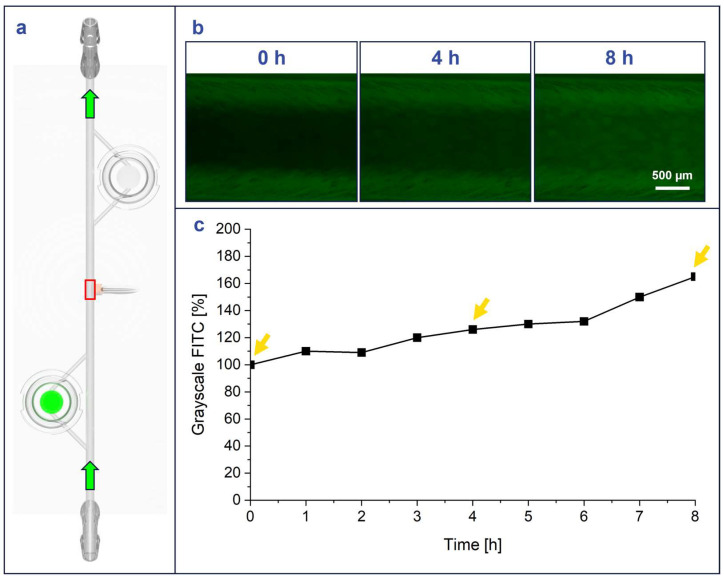
FITC-dextran flow from FITC-dextran laden CANA module shows increasing fluorescence in MiFD over time. (**a**) Position of FITC-dextran module (green disc) and measurement point on the device (red square), green arrows: flow direction. (**b**) Intensity-adjusted images in the main channel of the device at different time points (green fluorescence channel images). (**c**) Indexed grayscale values for the green channel (FITC) of images, yellow arrows indicating imaging time points in 5b.

A flow experiment was conducted with FITC-dextran, where FITC-dextran-laden agarose modules were loaded into the MiFD to visualize the flow of fluorescence out of the modules and the general flow throughout the system, as described in Section 2.6. (Figure 5a). The change in fluorescent signal coincided with the flow direction, as the perfused-DPBS carried the FITC-dextran from the agarose modules to the rest of the MiFD system. The results show that the MiFD functioned as intended, as the fluorescence signal decreased within the modules and increased at the sampling port in the middle of the MiFD over time (Figure 5b). The visual results were quantified by indexing the grayscale FITC signal inside the channel at t = 0 h compared to t = 4 h and t = 8 h, which measured a steady increase from 100% to 126% and 165%, respectively (Figure 5c).

### 3.4. Microscopy

**Figure 6:**
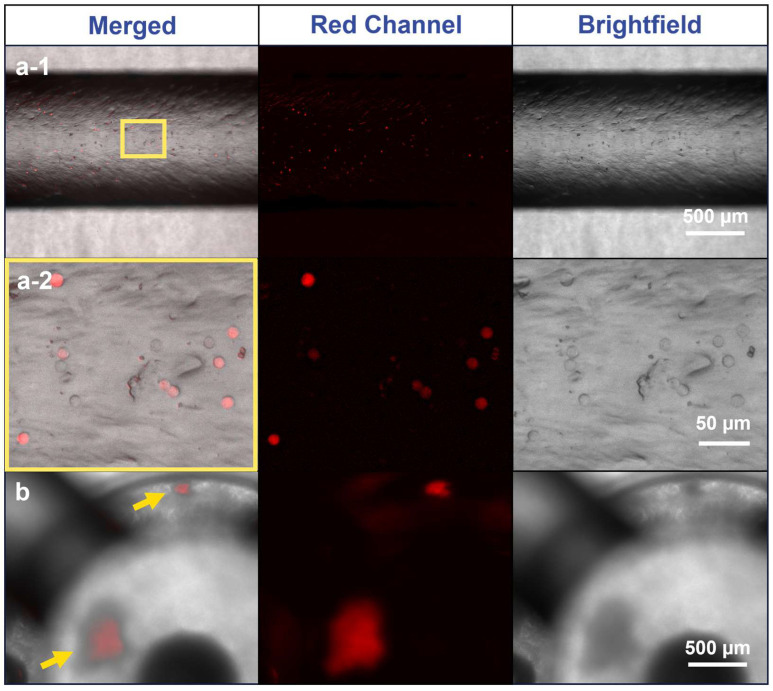
Fluorescence imaging of A549-RFP cells in the main channel and module of the MiFD. (**a-1**) Images of the main channel, yellow rectangle: segment for magnification in (**a-2**). (**a-2**) Images of cells in higher magnification. (**b**) Images of spheroids in an MiFD module, yellow arrows: indicating spheroid position. Background subtraction function applied with Fiji/ImageJ, original image in Figure A5.

**Figure A4:**
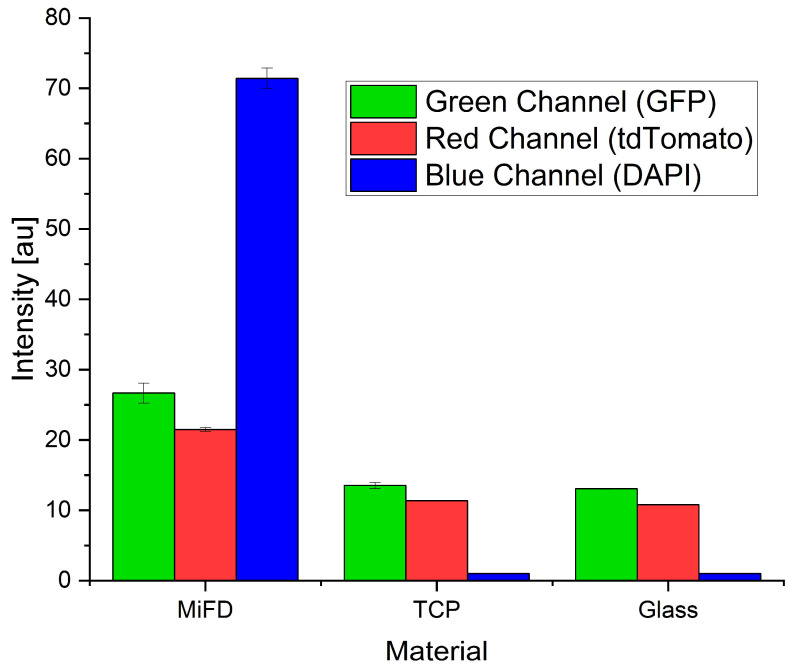
Autofluorescence of the MiFD, tissue culture plastic (TCP), and glass. Exposure time for all samples 200 ms. Intensity scale from 0 to 255.

**Figure A5:**
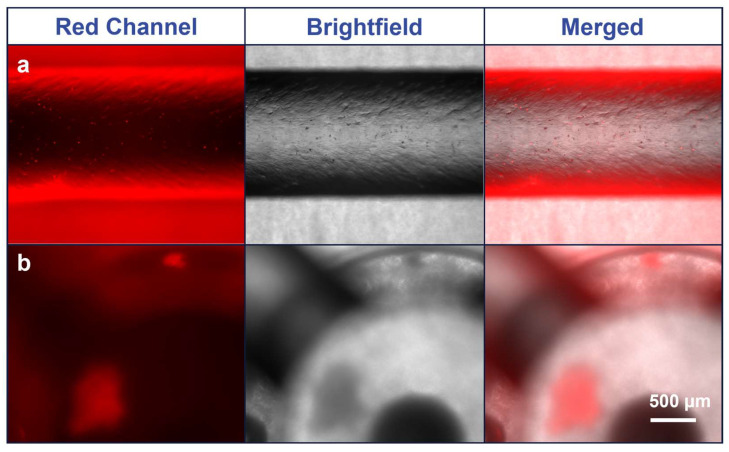
Fluorescence imaging of A549-RFP cells without background subtraction. (**a**) Fluorescence images of cells in the main channel. (**b**) Fluorescence images of spheroids in a module.

The Formlabs BioMed Clear resin appeared to have a degree of autofluorescence, and optical diffusion from the printed module surface and agarose inlay hindered precise imaging. However, fluorescence intensity measurements indicated the red channel had the lowest autofluorescence (Figure A4), allowing discernable imaging of A549-RFP spheroids and individual cells within the MiFD, as can be seen in Figure 6b. Although imaging cells directly in the modules proved challenging, cell visualization in the channels was straightforward. While pipetting a cell suspension through the MiFD, it was possible to discern the individual A549-RFP cells flowing through the system, as shown in Figure 6(a-1,a-2), with background subtraction. The original images can be seen in Figure A5.

**Figure A6:**
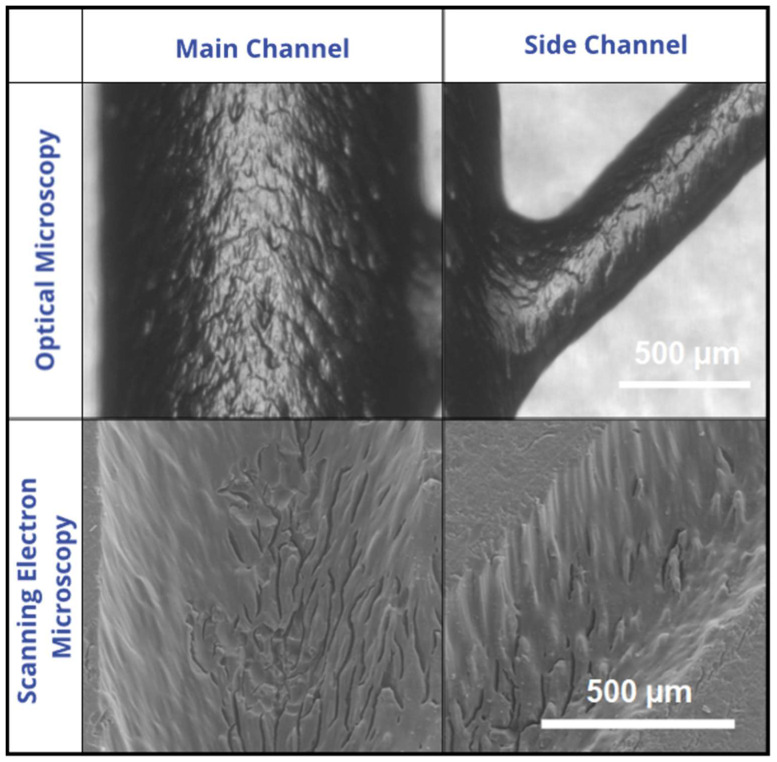
OM and SEM image comparison of channel surfaces. OM images captured through an intact device; SEM images captured of a gold-sputtered cross-sectioned device, detailing microscale surface topography.

Although cell seeding in the channels was not a focus of this study, microscopic inspection during the cellular flow test revealed surface irregularities, prompting further investigation via SEM. Comparative images between SEM and optical microscopy (OM) reveal a visibly rough surface (Figure A6).

### 3.5. Cytocompatibility of Bio-Resin

**Figure 7:**
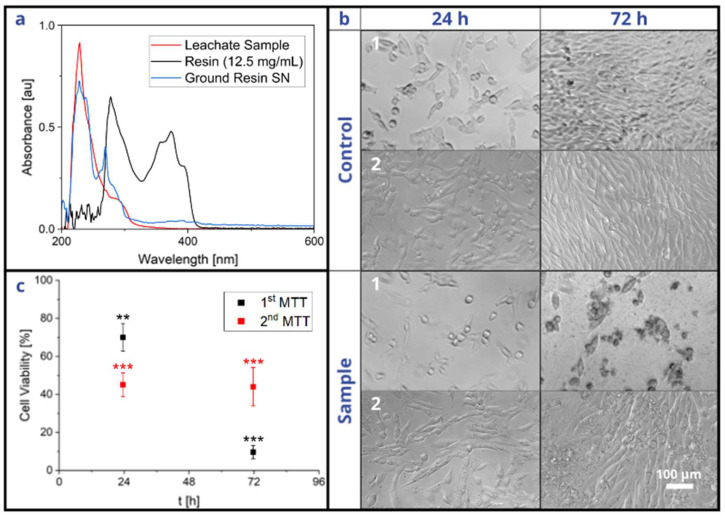
Leachate testing of the MiFD and its cytotoxicity. (**a**) Comparison of absorbances between a leachate sample, liquid resin, and supernatant from ground, printed resin. (**b-1**) Optical microscopy images of leachate-treated and untreated NIH/3T3 cells from a standard device. (**b-2**) Optical microscopy images of leachate-treated and untreated NIH/3T3 cells from an additionally washed device. (**c**) Viability of leachate-treated cells compared to controls from both standard and washed devices. Leachate extraction as described in the materials and methods section. Statistics with n = 3 each, ** *p* < 0.01; *** *p* < 0.001 significant differences between treated and untreated conditions at the same time point in a Student’s *t*-test.

When comparing the peaks of the leachate, ground solid resin supernatant, and liquid monomer resin, described in Section 2.7, it can be deduced that the leachate mainly consisted of polymerized resin rather than residual monomers, since those peaks closely overlap (Figure 7a). The results of the MTT assay, described in Section 2.8, show that the resin had a growth-inhibiting effect on the cells, as depicted in Figure 7b,c. After 24 h, there was little difference between the phenotypes of the control and sample groups. However, after 72 h, the control cells had multiplied to 100% confluency, whereas the sample cells had stopped propagating and were mostly detached from the substrate. Additionally, the MTT assay revealed that the sample cell viability fell below 70% after 72 h, which suggests that the polymerized resin has a potentially cytotoxic effect [39], even after printing and post-processing according to the standard operating procedures and despite its advertised biocompatibility. The second MTT assay, performed after a series of washing steps, also revealed significant cytotoxicity. However, the viability did not drastically decrease between 24 h and 72 h.

## 4. Discussion

Many recent publications focus on the utilization of µFDs. However, fabricating devices genuinely in the micrometer range is rather difficult, expensive, and labor-intensive [20,21,24]. Meanwhile, millifluidic and sub-millifluidic devices have already been shown to be sufficient for multiple applications [24] and can avoid the majority of the issues associated with true microfluidics, such as bubble formation, which can disrupt an experiment [16]. “Moreover, millifluidic devices with channel dimensions on the scale of the millimeter are less sensitive to clogging and fouling” [16] and are therefore also more ideal for 3D-printing methods [16]. A further advantage of millifluidics combined with 3D printing is the avoidance of traditional microfabrication, which is often resource-intensive, costly, and reliant on specialized equipment [24,25,26,27,28], making them unconducive to rapid prototyping and iterative design, which can severely limit research progress and development in studies [17,20]. Several PDMS demolding techniques have been demonstrated with 3D-printed molds and sacrificial filaments to bypass the costs and challenges of soft lithography and micromachining [30,43,44]. However, casting PDMS over 3D-printed molds yields only 2.5D features at best, and the requirement to remove a solid or semi-solid filament mold through the channels significantly limits channel interconnectivity, module design, and 3D geometry. In contrast, direct 3D printing enables the production of complex geometric matrices and modules with high design precision. Additionally, 3D printing technology allows for the sharing of cost-efficient design concepts [26], as demonstrated by the SLA-printed peristaltic pump from Jönsson et al. [45] or the one used in this study [38].

However, multiple 3D-printing methods exist, such as PolyJet (PJ) and fused deposition modeling (FDM), each of which has advantages and disadvantages. For example, PJ printing is efficient and offers high precision, but it is one of the most expensive options [21,46] and has been reported to be labor-intensive and demanding in post-processing [21,25,46]. In comparison, FDM printing is efficient, simple, and low-cost [21,46,47], with a broad range of available materials, including biocompatible options [21,47]. However, it has the lowest resolution among other printing methods, making it less suitable for intricate features [47], and it often produces visible layer lines [47], which are sometimes not sealed, hindering microscopic analysis and requiring extra steps in post-processing [33,46].

In contrast, SLA printing, as applied in this study, can produce accurate and high-resolution features with minimal and uncomplicated post-processing, the most distinct details, and the smoothest surface finishes of all plastic 3D-printing technologies [21,24,46,47]. The production of an MiFD could be completed within 15 h, with the high-resolution printing taking the largest amount of time at about 12.5 h. However, printing with a lower resolution could reduce the print time to about 6.5 h or 3 h for 0.05 mm and 0.1 mm layer heights, respectively. Additionally, post-processing, which comprises 6 straightforward steps, as outlined in Section 2.2: washing (20 min), cleaning (10 min), drying (30 min), curing (1 h), and polishing (15 min), accounted for a total of less than 2.5 h. Furthermore, in terms of cost for quality, SLA printing is superior. The material cost for the final design of this study, at 10 mL of material, was 4.15 EUR, 1.61 EUR, and 2.38 EUR for the Formlabs BioMed Clear, Formlabs Clear V4, and 3Dresyns OD Clear Bio resins, respectively. In comparison, Macdonald et al. concluded that for similarly sized devices that the price per device is 2 USD for SLA printing, while FDM and PJ cost 0.1 USD and 4 USD, respectively, and that all of these options are still considerably less expensive than a new PDMS microfabricated device at ~215 USD [46].

SLA printing is not without certain drawbacks, however. For example, the 3Dresyns OD Clear BIO and Formlabs BioMed Clear resins used in this study often took on a slight yellow tint when printed, as is typical for SLA resins [26]. The tinting has been attributed to visible light absorptivity and diffraction in the resin by Bhattacharjee et al. [26]. However, this study identified heat, particularly from autoclave sterilization, as the primary cause. Additionally, the printed MiFDs sometimes assumed a minor upward deflection, which may have been caused by stress development within the part due to thermal expansion and contraction of the resin during polymerization [48]. However, not all resins exhibit such defects, as Sharma et al. observed no deformations, discolorations, or structural changes in their study of autoclaved resins [48].

Nevertheless, other sterilization methods could be tested, such as soaking the parts in ethanol or ethylene oxide (ETO) under UV light exposure. However, such disinfectants must come into direct contact with microorganisms for inactivation to occur [49], and it is unclear how this would affect the photopolymer resin. Furthermore, autofluorescence of the printed resin complicated fluorescent microscopy of the MiFD in this study. Autofluorescence is often an issue for many UV-curable materials [50,51], and most commercially available printers utilize a single wavelength laser (405 nm) [29], which restricts material options. However, it is possible to formulate SLA resins that exhibit very little to no autofluorescence [29,52].

Another obstacle that SLA printing faces is resin cytotoxicity. Unfortunately, “the biocompatibility of materials used in 3D printing is largely unknown [25]”, with most SLA resins being toxic [25,26,29,46] and many even being labeled as biocompatible without specifying the intended use or which standards and certifications apply [26,53]. Indeed, even the definition of “biocompatibility” appears ambiguous across different resin manufacturers [53], and the definition may be based on various endpoints [51]. This ambiguity may lead users to perceive that they are compliant with the necessary standards without fully understanding the limitations of their selected material [53]. For example, in contrast to 3Dresyns, Formlabs provides clear instructions for using their dental resins, with suggestions for optimizing physical properties and biocompatibility [36] in addition to its hazards and limitations [54]. The BioMed Clear data sheet and the printing instructions suggest that the resin can be utilized for medical devices and device components, drug delivery devices, and research and development, among other uses, citing an assortment of ISO standards [36,55]. However, despite these claims, the BioMed Clear resin, along with the Form Wash and Form Cure, is also listed in a declaration of non-medical devices, as defined in Article I of the European Union’s Medical Device Directive (93/42/EEC) [56].

Furthermore, as shown in the results of this study, the Formlabs BioMed Clear resin exhibits cytotoxic potential, despite its biocompatible certifications and adherence to various ISO standards [55]. This cytotoxicity may be attributed to residual uncured resin within the printed components. As such, extended curing times could potentially improve biocompatibility [26,57], but the user protocols should address this. Nevertheless, the recent rise in SLA printing’s popularity has driven efforts to create more biocompatible resins [24,26,53], which can be expected to clarify material formulations and overcome limitations with time.

In the future, 3D printing could potentially be integrated with artificial intelligence (AI) to optimize and refine product design processes [34,35]. It has already been shown that a text-based AI model like ChatGPT can interpret a short series of user inputs to produce precise models and even small-scale fluidics [34]. When combined with 3D scanning and augmented reality, this technology could enable the rapid creation of intricate, customized models tailored to specific functionalities without requiring CAD expertise from the user. For example, by integrating AI-driven modeling with knowledge of biological processes, such as inter-tissue flow dynamics and cell migration, as described by Li et al. [14], along with medium or hydrogel properties and signaling gradients, as discussed by Ahrens et al. [58], devices could be designed to replicate complex biological environments more accurately. Moreover, incorporating AI into the design process could also drastically reduce design lead time, reduce waste in production, and enable real-time comparative modeling and simulation [35]. While still in its infancy, AI-based 3D printing shows considerable promise in increasing automation and efficiency [34]. Although 3D printing may not be ideally suited for large-scale industrial production, it has proven invaluable in clinical applications for personalized medicine. In particular, SLA printing, with its high resolution, fast production speed, and relatively low cost, has become the most widely adopted 3D printing technology in dentistry [59]. Given these strengths and immense potential, 3D printing is an excellent choice for developing MiFDs and other products, with applications spanning chemistry, electronics, biomedical engineering, and small-scale fluidics [33,47].

## 5. Conclusions

This study demonstrates the significant potential of SLA 3D printing for disease research with a functional MiFD for cell observation and material biocompatibility assessment. The study highlights the efficiency, cost-effectiveness, and design flexibility of SLA 3D printing. However, it also reveals potential hindrances like biocompatibility, surface roughness, and autofluorescence. Nevertheless, as printers become more accessible with better print configurations and resins improve, these obstacles will likely be overcome, bridging the gap between laboratory research, industrial production, and commercial applications, enabling the production of personalized cancer treatment devices. Finally, the DIY nature of 3D printing will democratize science, allowing researchers worldwide to contribute to solving global issues, and by including active reactors and AI-based models for predicting patient-specific metastatic potential, 3D printing of small-scale fluidics may become the leading device fabrication method in disease research and other fields.

## Data Availability

All data are available in the paper. Raw data can be provided upon request.
